# Attachment style moderates partner presence effects on pain: a laser-evoked potentials study

**DOI:** 10.1093/scan/nsu156

**Published:** 2015-01-01

**Authors:** Charlotte Krahé, Yannis Paloyelis, Heather Condon, Paul M. Jenkinson, Steven C. R. Williams, Aikaterini Fotopoulou

**Affiliations:** ^1^Department of Psychology, Institute of Psychiatry, Psychology and Neuroscience, King's College London, London, UK,; ^2^Department of Neuroimaging, Institute of Psychiatry, Psychology and Neuroscience, King’s College London, London, UK,; ^3^Department of Psychology, University of Hertfordshire, Hertfordshire, UK, and; ^4^Research Department of Clinical, Educational and Health Psychology, University College London, London, UK

**Keywords:** social presence, social support, pain, attachment style, laser-evoked potentials

## Abstract

Social support is crucial for psychological and physical well-being. Yet, in experimental and clinical pain research, the presence of others has been found to both attenuate and intensify pain. To investigate the factors underlying these mixed effects, we administered noxious laser stimuli to 39 healthy women while their romantic partner was present or absent, and measured pain ratings and laser-evoked potentials (LEPs) to assess the effects of partner presence on subjective pain experience and underlying neural processes. Further, we examined whether individual differences in adult attachment style (AAS), alone or in interaction with the partner’s level of attentional focus (manipulated to be either on or away from the participant) might modulate these effects. We found that the effects of partner presence *vs* absence on pain-related measures depended on AAS but not partner attentional focus. The higher participants’ attachment avoidance, the higher pain ratings and N2 and P2 local peak amplitudes were in the presence compared with the absence of the romantic partner. As LEPs are thought to reflect activity relating to the salience of events, our data suggest that partner presence may influence the perceived salience of events threatening the body, particularly in individuals who tend to mistrust others.

## INTRODUCTION

Human experience is inextricably embedded within a social world, from being part of a wider society to forming close relationships with other individuals. A key function of social connection is the provision of help and support in the face of threat (Bowlby, 1997/1969; Coan, 2008). Beneficial effects of social support have been found regarding a range of threats to physical and psychological well-being (Uchino, 2006). Studies investigating the mechanisms by which social support affects well-being have mainly focused on neuroendocrine stress responses (Kikusui *et al.*, 2006). However, more recently, the emerging field of social cognitive neuroscience has begun to examine the central neural mechanisms associated with receiving social support (reviewed in Eisenberger, 2013). Several such studies have focused on the neural mechanisms mediating the effects of social support on pain.

Two studies primed concepts of social support by presenting participants in pain with photographs of different social partners and found that viewing photographs of the romantic partner reduced pain ratings relative to viewing pictures of strangers, acquaintances, or objects (Younger *et al.*, 2010; Eisenberger *et al.*, 2011). Neural activity which correlated with pain reduction in the partner photograph conditions was found in brain regions associated with signalling safety (the ventromedial prefrontal cortex; Eisenberger *et al.*, 2011) and reward (e.g. nucleus accumbens; Younger *et al.*, 2010). However, these studies did not test the effects of a social partner who was physically present during pain. To our knowledge, only one neuroscientific study has experimentally investigated the effects of a physically present partner, but in relation to the anticipation of pain rather than the experience of pain itself. Coan *et al.* (2006) measured neural activity while participants were holding the hand of their romantic partner or a stranger, or holding no hand, during the threat of impending electric shocks. Participants reported lowest unpleasantness feelings when holding their partner’s hand, and associated activation was found in brain regions implicated in the regulation of emotion (e.g. the dorsolateral prefrontal cortex and caudate nucleus).

This study aimed to go beyond the above insights by examining how the perception of experimentally administered noxious stimuli was influenced by the actual presence of one’s romantic partner. Moreover, while neuroimaging studies highlight that social support from close others may be beneficial in reducing pain, behavioural studies into the effects of supportive social presence on pain have revealed a more complex picture (Krahé *et al.*, 2013). Social presence has been found to attenuate (Brown *et al.*, 2003) or increase pain (McClelland and McCubbin, 2008). These mixed results suggest the need to study not only how specific social contextual factors may modulate pain and related neural responses but also how personality factors may interact with such contextual variables.

A key personality factor that may influence the effects of social presence on pain is adult attachment style (AAS). AAS describes individual differences in representational models of close relationships which originate from early interactions with caregivers, remain relatively stable across the lifespan (Waters *et al.*, 2000), and apply to adult romantic relationships (Hazan and Shaver, 1987). Differences in AAS are frequently conceptualized along dimensions of attachment anxiety and avoidance (Fraley *et al.*, 2000). Individuals high on the anxiety but low on the avoidance dimension are anxiously attached. They crave closeness but fear abandonment, while individuals high on the avoidance but low on anxiety dimension are avoidantly attached and find it difficult to trust and depend on their partner (Hazan and Shaver, 1987).

These ‘insecure’ attachment styles have been associated with increased pain in experimental (Meredith, 2013) and clinical settings (e.g. in labour; Costa-Martins *et al.*, 2014), and have been proposed to constitute a vulnerability factor for developing chronic pain (Meredith *et al.*, 2008). This may be due to potentially maladaptive coping strategies employed by more insecure individuals. Anxiously attached individuals engage in ‘hyperactivating’ strategies; they are overly attentive to potential threat and highly motivated to secure support from their partner (Shaver and Mikulincer, 2002). Conversely, avoidant individuals employ ‘deactivating’ strategies, which minimize the importance of potential threats, and aim to cope on their own rather than turn to their partner for support (Shaver and Mikulincer, 2002). A behavioural study examining the interaction of AAS with social presence on pain demonstrated that higher attachment avoidance predicted more pain when another person (a research confederate) was present *vs* absent (Sambo *et al.*, 2010). This suggests that AAS may determine the effects of social presence on pain. However, the neural processes underlying these interactive effects have not been addressed in research thus far. In this study, we therefore obtained neuroscientific and subjective measures to investigate whether AAS moderates the effects of the presence of the romantic partner on pain ratings and pain-related neural processing.

Moreover, we sought to examine whether AAS as a stable personality trait interacts with situational cues to affect pain intensity ratings and associated neural responses in the presence of others. In Sambo *et al.*’s (2010) study, higher attachment anxiety predicted less pain when the research confederate was perceived to have high *vs* low empathy for the participant. This indicates that the pain-enhancing impact of anxious attachment was attenuated by the presence of a highly empathic person. In a similar behavioural study, higher attachment avoidance predicted less pain in the presence of the romantic partner, but only when participants were made to believe their partner had high empathy for them (Hurter *et al.*, 2014). Although the latter study included the physical presence of the romantic partner, presence was not varied (i.e. the partner was always present).

In this study, we manipulated partner attentional focus as a situational feature that might interact with AAS to shape the effects of partner presence on pain ratings and associated neural processing. This aspect has not yet been examined in neuroscientific pain research, but a behavioural study in the context of stress showed that individuals felt more secure walking along a virtual cliff (a stress-inducing task) when their romantic partner was attentive *vs* inattentive to them (Kane *et al.*, 2012). However, this study did not examine whether these effects were influenced by differences in AAS. Given the vigilance for signs of support in anxiously attached individuals and the preference for coping alone in avoidantly attached individuals (see Mikulincer *et al.*, 2003), we reasoned that for anxiously but not avoidantly attached individuals, partner presence would attenuate pain more if the partner’s attention was focused on them rather than elsewhere.

Previous neuroscientific studies into the social modulation of pain have mainly used fMRI methods (see Eisenberger, 2013). However, in pain research, laser-evoked potentials (LEPs) have been widely studied (see Bromm and Treede, 1984; Legrain *et al.*, 2011). LEPs are neurophysiological measures of evoked brain responses time-locked to transient, noxious thermal stimulation. They relate to the activation of Aδ nociceptive fibres and reflect both nociception and cortical processing of noxious stimuli (Lee *et al.*, 2009). Two types of LEPs are commonly observed in relation to noxious stimuli: the first is an early negative deflection, peaking ∼160 ms post-stimulus onset and termed N1 (Kunde and Treede, 1993). Source localization and intracranial recording studies have shown that N1 mainly reflects activation in operculoinsular and primary somatosensory cortices (Garcia-Larrea *et al.*, 2003; Valentini *et al.*, 2012). It has been proposed that N1 relates to early sensory (nociceptive) processing preceding the conscious experience of pain (Lee *et al.*, 2009). The second type of LEPs comprises a biphasic complex peaking around 200–350 ms, termed N2–P2, whose underlying cortical generators primarily comprise the operculoinsular and anterior cingulate cortices (Bromm and Treede, 1984; Garcia-Larrea *et al.*, 2003). The N2–P2 complex has been shown to reflect the conscious experience or ‘perceptual outcome’ of the sensory processing captured by N1 (Lee *et al.*, 2009).

LEPs are modulated by social contextual factors such as empathy for another’s pain (Valeriani *et al.*, 2008), but studies have not yet investigated the effects of receiving social support on LEPs. Examining both types of LEPs provides the opportunity to disentangle the effects of social contextual variables on different stages of pain-related neural processing, especially in regard to the influence of top-down *vs* bottom-up factors. Theoretical proposals on the neural mechanisms of pain and interoceptive perception (Craig, 2002, 2009) have suggested that integrated activity in the anterior insula and anterior cingulate cortex may allow social contextual variables to regulate bottom-up nociceptive signals, which themselves are thought to be processed and integrated further down the neurocognitive hierarchy and particularly in primary somatosensory areas and the posterior insula. In relation to LEPs, social contextual factors should therefore affect the N2–P2 component, reflecting this particular cortical processing, rather than the N1 component, which has been shown to be driven by sensory input. To investigate this proposition, we recorded LEPs while the presence and attentional focus of the partner was varied and explored effects on both N1 and N2–P2 components.

Based on the available behavioural studies, we first hypothesised that higher attachment anxiety would predict lower pain ratings and N2–P2 amplitudes in the presence *vs* absence of the romantic partner, and conversely, that higher attachment avoidance would predict higher values on these measures in the presence *vs* absence of the romantic partner. Our second hypothesis was that higher attachment anxiety would predict lower values on pain-related measures if the partner was focusing on *vs* away from the participant’s pain, and conversely, that higher attachment avoidance would predict higher values on these measures if the partner was focusing on *vs* away from the participant’s pain.

## METHOD

### Participants

Thirty-nine heterosexual couples in a romantic relationship were recruited from King’s College London and were approached using university circular e-mails. We experimentally induced pain in the women (henceforth ‘participant’). Men served as the social partner (hereafter ‘partner’). Participants were included if they were right-handed, had been in their current relationship for over a year, did not have a history of psychiatric (e.g. clinical depression), medical (e.g. chronic pain) or neurological conditions (e.g. epilepsy) and did not have a history of substance abuse. Further, participants were included only if they had not taken any medication (including painkillers) on the day of testing. The mean age of participants and their partners was *M* = 25.87 years (s.d. = 5.17) and *M* = 27.15 years (s.d. = 5.96), respectively. On average, couples had been together for *M* = 46.74 months (s.d. = 35.37). Participants were predominantly British (66.67%), from other European countries (23.08%) or from outside of Europe (10.26%). The majority of participants indicated that they were white (82.05%) or Asian (12.82%). Ethical approval was obtained from King’s College London Psychiatry, Nursing and Midwifery Research Ethics Subcommittee.

### Design

Our within-subjects design comprised three experimental conditions: two partner-present conditions with attention focused on either the participant’s pain (*Partner focus*) or another participant’s pain (*Other focus*) and a third *Partner absence *condition. The potential moderating role of AAS on pain was assessed by examining attachment anxiety and avoidance dimensions as continuous predictor variables. Outcome measures were mean pain rating, and mean local peak amplitude and latency for N1, N2 and P2 LEP components.

### Procedure

Couples attended one experimental session lasting ∼90 min (see Supplementary Figure S1 for session layout). It was explained that the aim of the study was to examine the effects of partner empathy on pain (the empathy task below was part of this cover story). Couples provided informed consent and were familiarized with the laser equipment before proceeding to the experimental conditions.

The experiment consisted of three 10-min laser blocks. In one block, couples were informed that the partner would be rating his empathy for the participant while she received laser stimuli (the *Partner focus* condition). Partners were told that they would rate their empathy for participants in response to real-time information about the laser intensities participants were receiving (see Supplementary Figure S1 for a depiction of how this information was presented). In the other two blocks, the partner would be rating his empathy for two participants who had previously taken part in the experiment (by viewing information on the laser intensities they had received), while the participant received laser stimuli (the *Other focus *and *Partner absence *conditions). He would therefore be unable to pay attention to the participant during these blocks. For one of these previous participants, the partner would be in the testing room (the *Other focus* condition). For the other, we led couples to believe that due to a technical fault, the file for the previous participant would not load on the lab computer. The partner was therefore going to rate his empathy on a computer next door, and would be absent for this block (the *Partner absence* condition).

The order of conditions was counterbalanced across couples. During each laser block, participants’ EEG was recorded. Couples were prevented from viewing each other by means of a curtain and instructed not to communicate during the blocks to avoid biasing participants’ pain ratings. After the third block, participants completed manipulation checks before couples were fully debriefed and paid £30 for their participation.

### Materials

#### Pain induction method and laser blocks

Pain was experimentally induced through an infrared neodymium yttrium aluminium perovskite (Nd:YAP) laser (Electronical Engineering, Italy) with a 1340-nm wavelength. We set the spot diameter to 5 mm at the skin site and the pulse duration to 4 ms. The experimental intensity was set individually for each participant during familiarization with the laser to correspond to a rating of ‘8’ (out of a maximum 10) on the pain rating scale (see *Pain ratings*). This was set to achieve a clear, moderately painful (but always tolerable) sharp pinprick sensation that is associated with the activation of Aδ nociceptive fibres and the induction of the LEPs in the EEG (Lee *et al.*, 2009). Experimental laser intensities had a mean of 3.80 J (s.d. = .55). We applied laser stimuli to all dorsal digits on participants’ left hand, changing the stimulation site between consecutive applications. Participants’ hands were maintained at a constant temperature during the laser stimulation (as in Ronga *et al.*, 2013).

Each laser block consisted of 35 trials at participants’ experimental intensity. In addition, we included 15 distractor stimuli (rated as ‘0’ out of 10 during familiarization; *M* = 1.78 J, s.d. = .04) and intentionally varied (i.e. jittered) the onset of each laser stimulus to increase the unpredictability of the intensity and timing of the laser stimuli. Therefore, the duration of each trial varied from 10 to 14 s. Experimental and distractor stimuli were presented in a pseudorandom order.

#### EEG recording

EEG data were collected using a 16-channel Guger Technologies Medical Engineering GmbH (g.tec; Austria) elasticized cap with an active electrode system and recorded using the g.tec g.recorder software. Eleven electrodes were positioned on the scalp according to the international 10–20 system, namely along the midline (Fz, FCz, Cz, CPz, Pz) and the left and right temporal regions (T7, C5, C3, T8, C6, C4) to be able to record N1 and N2–P2 components (Treede *et al.*, 2003). The electrooculogram (EOG) was recorded by placing one electrode above and one below the right eye. EEG channels were referenced to the right earlobe during data acquisition. Three further electrodes (bilateral mastoids and the nose) were included for offline re-referencing. Data were sampled at 512 Hz. A notch filter was applied at 50Hz to eliminate power line noise and data were online filtered between 0.1 and 100 Hz. Although filtering out a large band of low frequencies may attenuate components, a 0.1Hz filter has been found to leave components unaffected (Kappenman and Luck, 2010) and high-pass filters up to 0.15 Hz have been used in LEP research (e.g. Bentley *et al.*, 2003). Further, the same filter was applied across experimental conditions, meaning that comparisons of the components across conditions were unaffected by our filter choice.

### Measures

#### Pain ratings

After each laser stimulus, participants rated the intensity of the stimulus on an 11-point scale ranging from 0 (*no pinprick sensation*) to 10 (*the worst pinprick sensation imaginable*). The scale was presented on a computer screen and participants silently entered their ratings using a numeric keypad. A mean pain rating was calculated for each condition by averaging the pain ratings for the 35 experimental trials in that condition.

#### Laser-evoked potentials (LEPs)

As outlined above, LEPs consist of early (< 800 ms) negative and positive evoked deflections of the electroencephalogram time-locked specifically to the stimulation of fast-conducting Aδ nociceptive fibres. We recorded the local peak amplitude and local peak latency of N1 and N2–P2 components; see *Plan of analyses *for details. LEPs comprise relatively large and clearly distinctive components that are classically measured using the peak amplitude and latency (Iannetti *et al.*, 2008; Lee *et al.*, 2009; Mouraux and Iannetti, 2009; Terhaar *et al.*, 2011; Valentini *et al.*, 2012). We specifically measured *local* peak amplitudes, which take into account the voltages surrounding the peak and thus avoid mistakenly identifying the rising edges of adjacent components as peaks (Luck, 2014). We recorded the N1 component from the temporal region on the contralateral side to the laser stimulation site (i.e. C6 electrode), referenced to the Fz electrode. The negative-positive N2–P2 component was recorded from the vertex (Cz) electrode referenced to the average of both mastoid electrodes.

#### Adult attachment style (AAS)

The Experiences in Close Relationships Revised questionnaire (ECR-R; Fraley *et al.*, 2000) was used to measure AAS. This 36-item self-report measure of AAS yields continuous scores on attachment anxiety (18 items, e.g. ‘I often worry that my partner will not want to stay with me’; from 1 = *strongly disagree* to 7 = *strongly agree*) and attachment avoidance (18 items, e.g. ‘I find it difficult to allow myself to depend on romantic partners’) dimensions. Lower scores denote greater attachment security and higher scores greater attachment insecurity. Cronbach’s alpha was α = 0.83 for attachment anxiety and α = 0.94 for attachment avoidance.

#### Manipulation checks

Six statements assessed the success of our partner attentional focus manipulation: ‘When my partner was rating empathy for my/a previous participant’s pain, I felt he was paying attention to my pain’, ‘When my partner was rating empathy for my/a previous participant’s pain, I felt like his focus was mainly on me’, and ‘My partner rating his empathy for my/a previous participant’s made me pay more attention to my own pain’. Participants made their responses on a scale from 0 (*not at all*) to 7 (*extremely*). Responses to the three statements for ‘my pain’ (*Partner focus* condition) and ‘a previous participant’s pain’ (*Other focus* condition) were averaged separately and compared using a paired samples *t*-test.

### Plan of statistical analyses

To compare the effects of partner presence *vs* absence on our outcome measures, we created an overall ‘presence’ score by averaging (separately for each outcome measure) the data from *Partner focus* and *Other focus* conditions for each participant. To examine the effects of partner attentional focus, we compared *Partner focus* and *Other focus* conditions, excluding the *Partner absence* condition from these analyses.

All statistical analyses were conducted in Stata 13 (StataCorp, 2013). As repeated measures (Level 1) were nested within individuals (Level 2), multilevel modelling was implemented. We specified multilevel models with condition (either ‘presence/absence’ or ‘partner focus/other focus’) as a categorical predictor, attachment anxiety and attachment avoidance as continuous predictors, and included all interaction terms. Continuous predictors were centered around the mean prior to inclusion in the models to avoid multicollinearity issues otherwise problematic in regression-based models (Tabachnick and Fidell, 2007). In addition, we controlled for any effects of age, length of relationship and depression severity (see Supplementary Materials) as these were related to AAS scores. Significant interactions were followed up using the Stata ‘margins’ and ‘lincom’ commands to examine differences between conditions at low (−1 s.d.), moderate (mean) and high (+1 s.d.) continuous AAS scores. Cohen’s ƒ^2^ effect size was calculated for significant effects as appropriate for multilevel modelling analysis (Selya *et al.*, 2012).

Analyses were conducted separately for pain ratings and N1, N2 and P2 local peak amplitude and latency outcomes (as in e.g. Truini *et al.*, 2010). To correct for multiple testing, we used the ‘simes’ method (a method to correct for false discovery rate; Newson, 2003) in Stata 13 to calculate the critical *P* value used to evaluate statistical significance. Using this method, the critical *P* value was *P* = 0.015.

#### EEG processing

EEG data was processed using the open source toolboxes EEGLAB and ERPLAB for MATLAB (Matlab and statistics toolbox release R 2011a). The data were downsampled to 256Hz, offline bandpass filtered between 0.4 and 30 Hz (a relatively high high-pass filter is typically used in LEP research; Lee *et al.*, 2009; Ronga *et al.*, 2013), segmented into −200 to 800 ms epochs in relation to stimulus onset, and baseline corrected using the 200-ms window before stimulus onset. Trials with muscle and eye blink artefacts were rejected using moving-window-to-peak analysis. Averaged potentials were calculated for the experimental stimuli trials only. We measured N1 and N2–P2 component local peak amplitudes and latencies for individual blocks where at least 70% (25 trials) of experimental trials remained after artifact rejection. The ERPLAB measurement tool was used to measure local peak amplitude and latency for N1, N2 and P2 separately. ERPLAB takes into account the voltages surrounding the peak in calculating local peak amplitudes (Luck, 2014). N1 was defined as the most negative peak in a time window of 0–270 ms post stimulus onset. N2 was operationalized as the most negative peak occurring in a 0–350 ms time window after stimulus onset and P2 was defined as the most positive peak in a 0–600 ms time window following stimulus onset.

One participant was excluded because no EEG data were available due to a fault during EEG recording. Furthermore, three participants were excluded from N1 and two from N2–P2 analyses because they had no averaged potentials on any of the three experimental conditions. In addition, four participants were excluded from N1 and one participant from N2 analyses because the ERPLAB measurement tool failed to return plausible local peak latency values (i.e. it returned values < 100 ms; previous studies have reported the earliest neural activity associated with laser stimulation to occur from 120 ms; Valentini *et al.*, 2012) on all three conditions. As missing data can be estimated in multilevel modelling (Gueorguieva and Krystal, 2004; Tabachnick and Fidell, 2007), N1, N2 and P2 local peak amplitudes and latencies were estimated using maximum likelihood estimation for participants for whom averaged potentials were available and for whom the ERPLAB measurement yielded plausible components in at least one condition. The instances where particular components could not be plausibly identified were randomly distributed across the data, indicating noise in the EEG recording rather than a systematic bias and fulfilling the criterion for estimation in multilevel modelling (Gueorguieva and Krystal, 2004). Overall, *n* = 31 participants were retained in N1 analyses, *n* = 35 in N2 analyses and *n* = 36 in P2 analyses.

## RESULTS

### Descriptive statistics

Mean AAS scores were *M* = 2.30 (s.d. = 0.73) for attachment anxiety and *M* = 2.25 (s.d. = 1.03) for attachment avoidance. Comparing this to American ECR-R norms, participants fell between the 20th and 30th percentile for attachment anxiety and the 30–40th percentile for attachment avoidance (R. Chris Fraley, personal communication, 2011). AAS scales were correlated at *r* = 0.59, *P* < 0.05. Mean pain ratings and mean values for N1, N2 and P2 local peak amplitude (LPA; µV) and local peak latency (LPL; ms) are presented in Table 1. Local peak amplitudes and latencies were in line with values previously reported in the literature (Treede *et al.*, 2003; Ronga *et al.*, 2013).

**Table 1:** Means and standard deviations for all outcome variables for the three experimental conditions and the averaged presence condition

		Partner focus		Other focus		Partner absence		Presence (average of Partner focus and Other focus)	
		Mean	s.d.	Mean	s.d.	Mean	s.d.	Mean	s.d.
Mean pain rating		4.69	1.35	4.59	1.56	4.56	1.35	4.64	1.35
N1	LPA (µV)	−9.29	3.98	−9.17	3.75	−10.45	5.10	−9.45	2.33
	LPL (ms)	177.47	21.66	177.67	19.79	176.76	18.26	178.31	16.41
N2	LPA (µV)	−13.55	6.77	−13.41	7.60	−13.98	7.76	−14.02	6.54
	LPL (ms)	211.12	19.61	208.01	19.98	210.05	23.53	207.98	18.14
P2	LPA (µV)	24.89	9.27	24.43	9.02	23.39	9.52	25.76	8.26
	LPL (ms)	360.09	50.38	362.61	56.19	354.48	44.33	358.62	47.56

*Notes*. LPA = local peak amplitude; LPL = Local peak latency.

### Multilevel modelling results

#### Does AAS moderate the effects of partner presence on pain and associated neural responses?

Full model results for partner presence analyses are presented in Table 2. A significant main effect of partner presence was found for P2 local peak amplitude, which was significantly higher in the presence (*M* = 24.74 µV, s.d. = 1.49) compared to the absence (*M* = 22.90 µV, s.d. = 1.44) condition, *b* = 3.28, SE = 1.13, *P* = 0.004, but not for any other outcome measures. Regarding main effects of attachment anxiety, higher attachment anxiety predicted a shorter latency to the laser stimuli for N1, *b* = −14.52, SE = 5.31, *P* = 0.006 (see Supplementary Figure S2), and N2 components, *b* = −20.49, SE = 5.49, *P* < 0.001; no other main effects were significant (see Table 2). Regarding attachment avoidance, one main effect reached significance: higher attachment avoidance predicted a smaller N2 local peak amplitude, *b* = 4.80, SE = 1.69, *P* = 0.005.

**Table 2:** Partner presence vs absence: multilevel modelling results for all outcome measures, controlling for participant age, length of relationship and depression severity

Effect	Dependent variable		Unstandardized coefficient (*b*)	Standard error	*P* value (critical value = 0.015)	95% confidence interval	
						Lower	Upper
Partner presence *vs* absence	Pain rating		0.12	0.12	0.315	−0.11	0.35
	N1	LPA	0.99	0.85	0.240	−0.67	2.65
		LPL	−0.04	2.17	0.985	−4.30	4.22
	N2	LPA	−0.69	0.88	0.435	−2.40	1.03
		LPL	−1.23	2.44	0.616	−6.02	3.56
	P2	**LPA**	**3.28**	**1.13**	**0.004**	**1.06**	**5.49**
		LPL	−1.09	6.93	0.875	−14.67	12.50
Attachment anxiety	Pain rating		0.74	0.37	0.043	0.02	1.46
	N1	LPA	2.06	1.32	0.118	−0.52	4.65
		**LPL**	−**14.52**	**5.31**	**0.006**	−**24.94**	−**4.10**
	N2	LPA	−2.54	2.14	0.236	−6.73	1.66
		**LPL**	−**20.49**	**5.49**	**0.000**	−**31.25**	−**9.72**
	P2	LPA	0.69	2.63	0.792	−4.46	5.85
		LPL	−21.05	12.49	0.092	−45.53	3.43
Attachment avoidance	Pain rating		−0.46	0.25	0.073	−0.97	0.04
	N1	LPA	−2.92	1.26	0.021	−5.39	−0.45
		LPL	5.27	5.11	0.303	−4.76	15.30
	N2	**LPA**	**4.80**	**1.69**	**0.005**	**1.48**	**8.12**
		LPL	5.85	4.36	0.180	−2.70	14.40
	P2	LPA	−1.87	2.00	0.349	−5.78	2.04
		LPL	14.39	9.60	0.134	−4.42	33.20
Partner presence × attachment anxiety	Pain rating		−0.17	0.18	0.367	−0.53	0.19
	N1	LPA	−0.23	1.20	0.850	−2.59	2.13
		LPL	−0.73	3.05	0.809	-6.71	5.24
	N2	LPA	1.85	1.30	0.154	−0.70	4.39
		LPL	5.94	3.62	0.101	−1.15	13.04
	P2	LPA	0.14	1.67	0.934	−3.13	3.41
		LPL	−3.64	10.26	0.723	−23.75	16.47
Partner presence × attachment avoidance	Pain rating		**0.35**	**0.13**	**0.007**	**0.10**	**0.61**
	N1	LPA	−0.30	1.22	0.803	−2.68	2.08
		LPL	1.15	3.20	0.718	−5.11	7.42
	N2	**LPA**	−**2.60**	**1.06**	**0.014**	−**4.67**	−**0.53**
		LPL	−4.96	2.95	0.092	−10.73	0.81
	P2	**LPA**	**3.29**	**1.32**	**0.013**	**0.71**	**5.87**
		LPL	−14.01	8.12	0.084	−29.92	1.90
Attachment anxiety × attachment avoidance	Pain rating		0.01	0.22	0.981	−0.42	0.44
	N1	LPA	1.57	0.83	0.058	−0.05	3.20
		LPL	−0.47	3.35	0.889	−7.03	6.10
	N2	LPA	−2.42	1.28	0.058	−4.92	0.08
		LPL	−2.86	3.27	0.382	−9.28	3.56
	P2	LPA	2.90	1.53	0.057	−0.09	5.90
		LPL	−9.92	7.25	0.171	−24.14	4.30
Partner presence × attachment anxiety × attachment avoidance	Pain rating		−0.08	0.11	0.484	−0.29	0.14
	N1	LPA	−0.36	0.69	0.596	−1.71	0.98
		LPL	−1.08	1.78	0.543	−4.57	2.41
	N2	LPA	2.67	1.78	0.133	−0.81	6.16
		LPL	−0.83	4.93	0.867	−10.49	8.83
	P2	LPA	−1.88	2.29	0.411	−6.37	2.60
		LPL	8.04	13.65	0.556	−18.72	34.80

*Note*. Significant results are highlighted using bold font. LPA = local peak amplitude; LPL = Local peak latency.

**Fig. 1:**
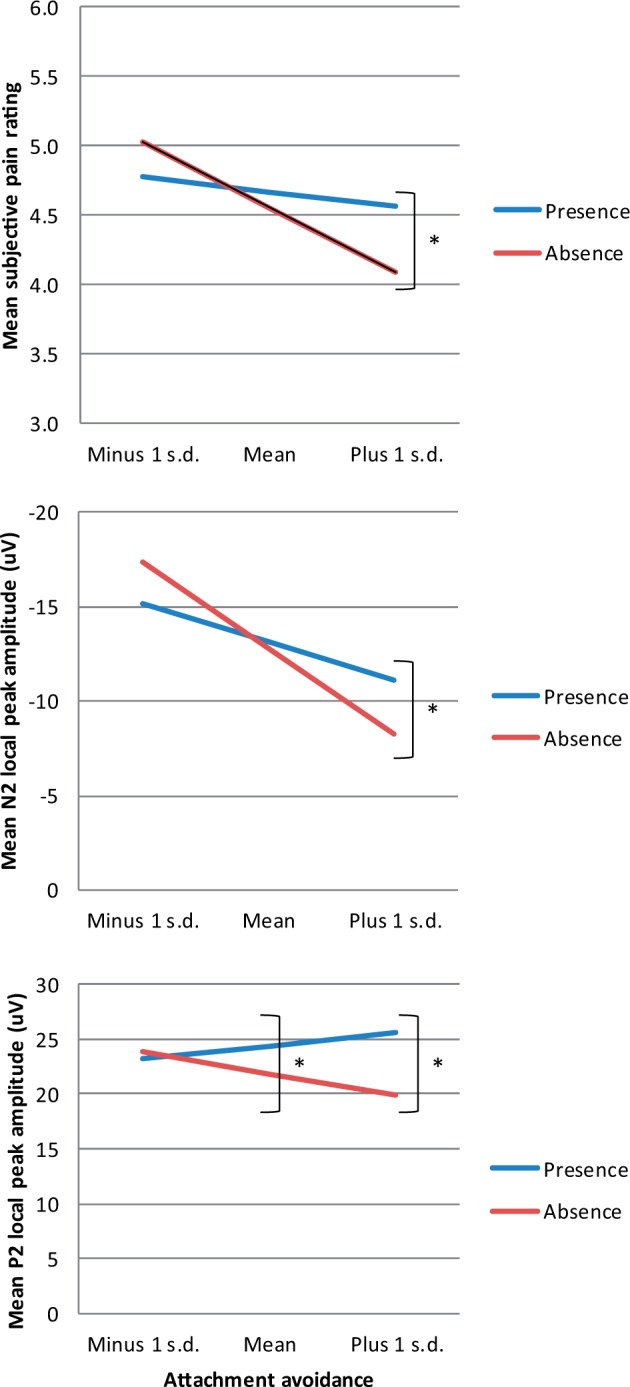
Partner presence by attachment avoidance interaction effects for pain rating (top panel), N2 local peak amplitude (middle panel) and P2 local peak amplitude (bottom panel). Statistically significant differences are marked by asterisk.

The hypothesised partner presence by attachment anxiety interaction was not significant for any outcome measures (see Table 2). However, partially supporting our first hypothesis, we found a significant partner presence by attachment avoidance interaction on pain rating, and on N2 and P2 local peak amplitude. The interaction effect on pain rating, *b* = 0.35, SE = 0.13, *P* = 0.007, ƒ^2 ^= 0.185, is presented in the top panel of Figure 1. Follow-up tests showed that the difference between presence and absence was significant for high attachment avoidance, *b* = −0.48, SE = 0.19, *P* = 0.011, but not for moderate, *b* = −0.11, SE = 0.11, *P* = 0.324, or low attachment avoidance, *b* = 0.25, SE = 0.17, *P* = 0.131. The significant partner presence by attachment avoidance interaction on N2 local peak amplitude, *b* = −2.60, SE = 1.06, *P* = 0.014, ƒ^2 ^= 0.334, is displayed in the middle panel of Figure 1 (see also Supplementary Figure S3). The difference between presence and absence was significant for high attachment avoidance, *b* = 2.93, SE = 1.44, *P* = 0.043, but not moderate, *b* = 0.35, SE = 0.84, *P* = 0.676, or low avoidance, *b* = −2.22, SE = 1.14, *P* = 0.051. The significant partner presence by attachment avoidance interaction on P2 local peak amplitude, *b* = 3.29, SE = 1.32, *P* = 0.013, ƒ^2 ^= 0.371, is presented in the bottom panel of Figure 1 (see also Supplementary Figure S3). The difference between presence and absence was significant for high, *b* = −5.67, SE = 1.82, *P* = 0.002, and moderate, *b* = −2.49, SE = 1.06, *P* = 0.019, but not low attachment avoidance, *b* = 0.70, SE = 1.44, *P* = 0.626.

In sum, consistent with our first hypothesis, the higher the attachment avoidance, the higher the pain rating, N2 and P2 local peak amplitude were during partner presence compared to partner absence. The interaction between partner presence and attachment anxiety was non-significant for all outcome measures. Furthermore, there was no significant two-way interaction between attachment dimensions and no three-way interaction of partner presence, attachment anxiety and attachment avoidance on any outcome measures (see Table 2), indicating that the results were driven by the attachment avoidance dimension.

#### Does AAS interact with partner attentional focus to shape partner presence effects on pain and associated neural responses?

Participants indicated that they felt their partner focused more on them in the *Partner focus *(*M* = 4.21, s.d. = 1.38) than the *Other focus* condition (*M* = 1.84, s.d. = 1.36), *t*(38) = 8.44, *P* < 0.001, supporting our partner attentional focus manipulation. Full model results for partner attentional focus analyses are presented in Supplementary Table S1. Using the adjusted critical *P* value, we did not find significant interactions of partner attentional focus with either AAS dimension for any of the outcome variables. Therefore, our second hypothesis was not supported.

## DISCUSSION

A fundamental function of connecting with others is their ability to provide support and security in the face of threat, such as pain. While priming social support has led to pain-attenuating effects and corresponding modulation of neural responses (Eisenberger, 2013), experimental investigations into social presence effects on pain have yielded mixed results (Brown *et al.*, 2003; McClelland and McCubbin, 2008). In this study, we examined the neural mechanisms underlying the effects of the physical presence of the romantic partner on pain and investigated whether individual differences in AAS, alone or in interaction with the partner’s degree of attentional focus, might explain some of this variability. We investigated the interacting effects of these social variables on pain-related neural responses, namely LEPs, associated both with nociception and cortical pain-related processing.

Consistent with our theoretical reasoning, we found that partner presence effects were shaped by AAS. In particular, attachment avoidance moderated the effects of partner presence on pain-related outcomes. Higher attachment avoidance predicted higher pain ratings and N2 and P2 amplitudes during the presence compared to the absence of the romantic partner. This finding extends Sambo *et al.*’s (2010) results by showing that partner presence (rather than the presence of an unfamiliar confederate) interacted with attachment avoidance to affect not only subjective ratings but also associated neural processing. LEPs have recently been proposed to reflect neural processing that is not necessarily pain-specific, but relates more generally to salient sensory events that may alert the body to threat in its environment (Legrain *et al.*, 2011). Indeed, laser stimuli are more likely to be classified as painful when participants believe them to be threatening rather than safe, and this is associated with activity in neural regions processing salient events, notably the anterior insula (Wiech *et al.*, 2010).

Our findings suggest that the noxious stimuli administered were more salient and possibly indicated a greater threat when the partner was present in conjunction with higher attachment avoidance. Avoidant individuals tend to hold negative perceptions of social support (Collins and Feeney, 2004). They prefer to deal with threat on their own and are less likely to turn to their support network than secure or anxious individuals (Ognibene and Collins, 1998; Wallace and Vaux, 1993). Thus, the unwanted presence of their partner may interfere with avoidant individuals’ coping strategies, including their aim to ‘inhibit the experience of aversive emotional states and exclude these states from awareness’ (Mikulincer *et al.*, 2003, p. 88). In this study, partner presence may have reduced the inhibition of pain-related neural processing. This may have encouraged noxious stimuli to reach consciousness and maintain their salience, thus warning individuals of the possible threat they were attempting to inhibit. Relating to this, we did not find any main or interaction effects of partner presence and attachment avoidance on N1, indicating that early sensory processing preceding conscious awareness was unaffected by these factors. This provides further support for the proposal that the influence of top-down social contextual factors on the experience of pain is modulated by the anterior insula and anterior cingulate cortex (Craig, 2009; Haggard *et al.*, 2013), both of which have been posited as main cortical generators of the N2–P2 component (see Garcia-Larrea *et al.*, 2003). Our findings will need to be replicated and examined within a clinical context; however, overall they provide preliminary evidence that social support during pain may need to be tailored to individual personality traits and coping preferences (see also Krahé *et al.*, 2014).

Evidence that attachment anxiety predicted reduced pain in the presence of an ostensibly highly empathic social partner (Sambo *et al.*, 2010) led us to hypothesize that attachment anxiety would predict attenuation of pain-related measures during partner presence. However, this was not supported by the data. A possible explanation is that the association between attachment anxiety and pain was too robust to be influenced by our temporary partner presence manipulation. In line with this possibility, higher attachment anxiety predicted faster neural responses (N1 and N2) to the laser stimuli across our experimental conditions. This is especially interesting regarding the early N1 component, which was unaffected by our social manipulations. Perhaps this brain response is not modulated by relatively transient social contextual factors (see above) but can be influenced by certain ingrained personality traits. The aforementioned hyperactivating coping strategies, involving ‘reacting quickly and vocally to early, and perhaps ambiguous, cues of imminent danger’ (Ein-Dor *et al.*, 2011, p. 80) might explain the relationship between attachment anxiety and N1 latency.

Contrary to our second hypothesis, AAS did not interact with partner attentional focus to influence pain, despite the manipulation checks indicating that perceived partner attentional focus varied between conditions as intended. It is possible that situational information about one’s partner’s attentional focus may have little weight as an indicator of social support over and above expectations of responsiveness formed in an existing romantic relationship. In the context of interactions with strangers, however, attentional focus may be a salient cue in shaping social presence effects. Indeed, effects of social support on pain depend strongly on the relationship between the person in pain and the social partner (see Krahé *et al.*, 2013). A future study could vary the level of attentional focus from both the partner and a stranger in the same experimental context to examine the influence of different interaction histories in conjunction with AAS on the pain experience.

This study had several limitations. To be able to selectively manipulate attentional focus as well as obtain unbiased pain ratings, we did not allow partners to have visual contact during the experimental blocks. Future research could aim to increase the salience of the focus manipulation by giving the present person an even more active role, e.g. the ability to terminate laser trials if they deem them to be too painful for the participant. Furthermore, our sample generally scored towards the lower end of both attachment dimensions. Therefore, our findings pertain to *relatively* high attachment anxiety and avoidance. However, the fact that we found such large effects despite our sample clustering towards the lower end of the attachment dimensions attests to the robustness of these findings. These large effects, in conjunction with our within-subjects design, also support the reliability of our findings, even though our sample size was relatively small (see Friston, 2012, for the relationship between sample and effect sizes). Nevertheless, future research could aim to replicate our results in larger samples of individuals pre-selected to score highly on one or the other attachment dimension. Lastly, previous studies have found participants’ AAS to interact with their partner’s AAS in influencing pain; for example, participants’ pain was highest when both they and their partner were characterized by an anxious AAS (Wilson and Ruben, 2011). Future research in couples could further examine the role of the partner’s AAS in shaping participants’ pain ratings and associated neural responses across a range of social conditions.

In conclusion, we found that the effects of partner presence on pain ratings and pain-related neural processing depended on individual differences in AAS and not the partner’s level of attentional focus. In particular, partner presence may not have beneficial effects on the experience of pain when the individual in pain is characterized by higher attachment avoidance. In planning future research into the neural mechanisms underlying the social modulation of pain, it therefore seems important to consider the moderating impact of AAS.

## SUPPLEMENTARY DATA

Supplementary data are available at *SCAN* online.

Supplementary Data
