# Supplementary material for: Attachment style moderates partner presence effects on pain: a laser-evoked potentials study
Source: Soc Cogn Affect Neurosci. 2015 Jan 1;10(8):1030–7. doi: 10.1093/scan/nsu156 (PMC4526477; doi:10.1093/scan/nsu156)
Supplement: Supplementary Data [file supp_10_8_1030__index.html]

Attachment style moderates partner presence effects on pain: a laser-evoked potentials study — Attachment style moderates partner presence effects on pain: a laser-evoked potentials study — Supplementary Data 

# Attachment style moderates partner presence effects on pain: a laser-evoked potentials study

## Supplementary Data

files

**Files in this Data Supplement:**

- Supplementary Data - docx file
