# Supplementary material for: Attachment style moderates partner presence effects on pain: a laser-evoked potentials study
Source: Soc Cogn Affect Neurosci. 2015 Jan 1;10(8):1030–7. doi: 10.1093/scan/nsu156 (PMC4526477; doi:10.1093/scan/nsu156)
Supplement: Supplementary Data [file supp_nsu156_scan-14-302-File006.docx]

**SUPPLEMENTARY MATERIAL**

*Depression severity*

The Patient Health Questionnaire Depression Module (PHQ-9; Kroenke & Spitzer, 2002) was used to control for depression severity in our analyses. The PHQ-9 is a 9-item self-report measure of depression providing both a diagnosis for Major Depressive Disorder (MDD) and Major Depressive Syndrome (MDS), and a depression severity score. Items are presented with the answer options *0 (not at all)*, *1 (several days)*, *2 (more than half the days)* and *3 (nearly every day)*, and are summed to calculate the total score (0 – 27). Diagnostic criteria include responding “2” on one of two specific items, and “2” on at least five further items for MDD and 2-4 further items for MDS.

Figure 1. *A schematic representation of the study procedure. Block order was counterbalanced across participants. Block structure and empathy task were identical for all blocks.*

Figure 2. *Main effect of attachment anxiety on N1 latency. For illustrative purposes, attachment anxiety has been divided into low and high attachment anxiety by means of a median split and the effect on the average of the three experimental conditions is displayed.*

Figure 3. *Partner condition (presence vs. absence) by attachment avoidance interaction on the N2-P2 component. For illustrative purposes, attachment avoidance is split into high and low avoidance by means of a median split. As the analyses reported in the paper were performed on continuous attachment scores, this figure may deviate slightly from the results reported in the paper.*

Table 1. *Partner focus vs. other focus: Multilevel modelling results for all outcome measures, controlling for participant age, length of relationship and depression severity.*


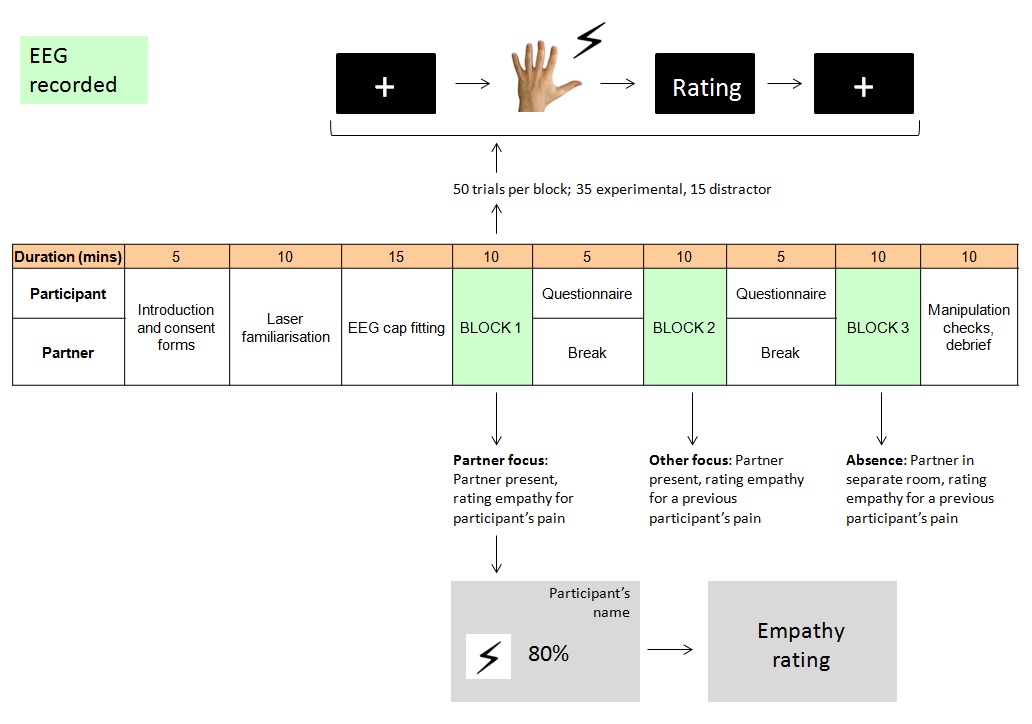


Figure 1. *A schematic representation of the study procedure. Block order was counterbalanced across participants. Block structure and empathy task were identical for all blocks.*


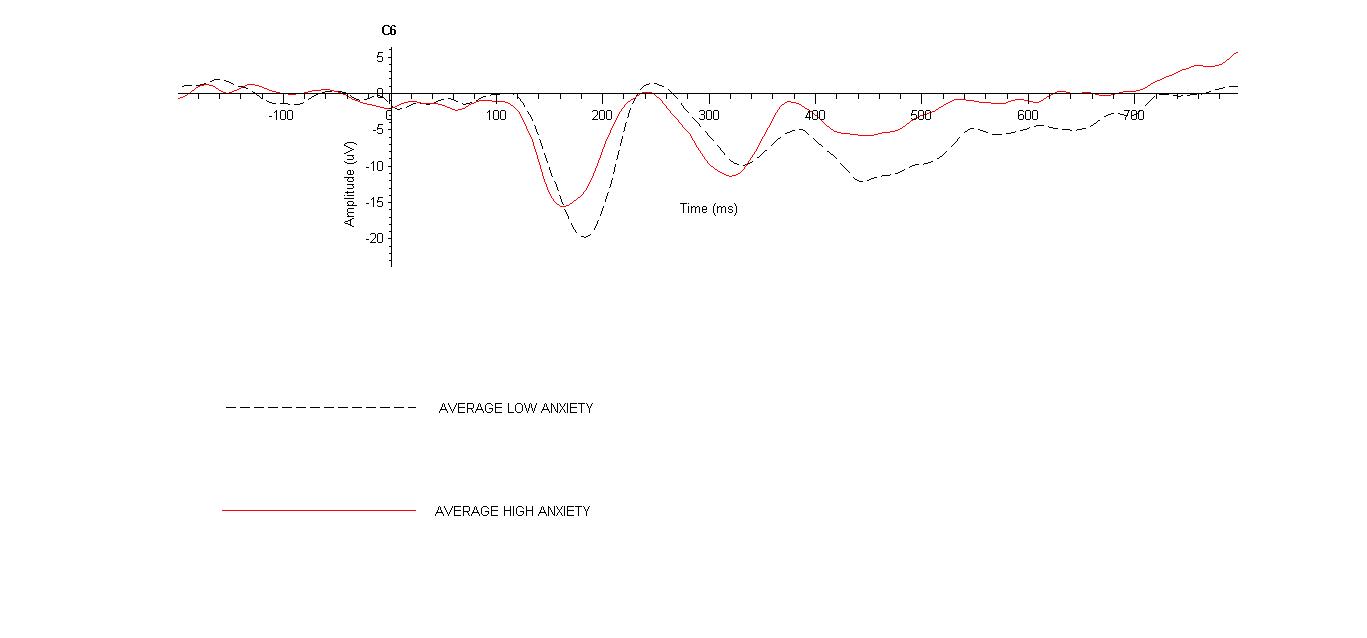


Figure 2. *Main effect of attachment anxiety on N1 latency. For illustrative purposes, attachment anxiety has been divided into low and high attachment anxiety by means of a median split and the effect on the average of the three experimental conditions is displayed.*


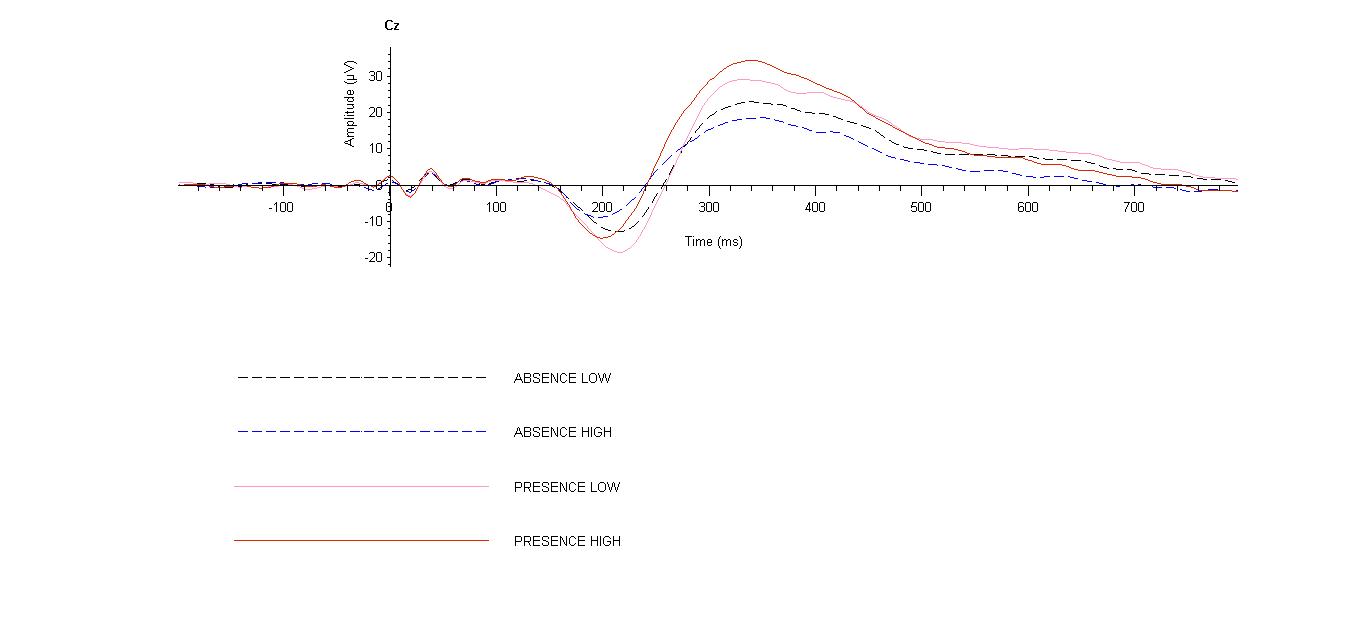


Figure 3. *Partner condition (presence vs. absence) by attachment avoidance interaction on the N2-P2 component. For illustrative purposes, attachment avoidance is split into high and low avoidance by means of a median split. As the analyses reported in the paper were performed on continuous attachment scores, this figure may deviate slightly from the results reported in the paper.*

Table 1. *Partner focus vs. other focus: Multilevel modelling results for all outcome measures, controlling for participant age, length of relationship and depression severity.*

| Effect | Dependent variable | | Unstandardised coefficient (b) | Standard error | p value (critical value = .015) | 95% confidence interval | |
| --- | --- | --- | --- | --- | --- | --- | --- |
|  |  |  |  |  |  | Lower | Upper |
| Partner focus vs. Other focus | Pain rating | | 0.16 | 0.19 | 0.390 | -0.21 | 0.53 |
|  | N1 | LPA | 0.77 | 0.94 | 0.413 | -1.07 | 2.60 |
|  |  | LPL | 4.51 | 3.23 | 0.163 | -1.82 | 10.84 |
|  | N2 | LPA | -0.37 | 1.29 | 0.777 | -2.90 | 2.17 |
|  |  | LPL | 4.80 | 3.06 | 0.117 | -1.20 | 10.81 |
|  | P2 | LPA | -0.26 | 1.20 | 0.828 | -2.62 | 2.10 |
|  |  | LPL | -1.39 | 8.00 | 0.862 | -17.07 | 14.29 |
| Attachment anxiety | Pain rating | | 0.86 | 0.40 | 0.032 | 0.07 | 1.64 |
|  | N1 | LPA | -1.00 | 1.23 | 0.417 | -3.40 | 1.41 |
|  |  | LPL | -12.27 | 5.85 | 0.036 | -23.73 | -0.82 |
|  | N2 | LPA | -0.43 | 2.13 | 0.839 | -4.61 | 3.74 |
|  |  | LPL | -11.88 | 5.22 | 0.023 | -22.10 | -1.66 |
|  | P2 | LPA | 0.88 | 2.69 | 0.743 | -4.39 | 6.15 |
|  |  | LPL | -27.30 | 14.32 | 0.057 | -55.36 | 0.76 |
| Attachment avoidance | Pain rating | | -0.25 | 0.28 | 0.369 | -0.80 | 0.30 |
|  | N1 | LPA | -0.63 | 1.08 | 0.562 | -2.74 | 1.49 |
|  |  | LPL | 1.20 | 5.10 | 0.814 | -8.80 | 11.20 |
|  | N2 | LPA | 2.65 | 1.69 | 0.116 | -0.66 | 5.97 |
|  |  | LPL | -1.05 | 4.14 | 0.799 | -9.16 | 7.05 |
|  | P2 | LPA | 1.17 | 2.06 | 0.570 | -2.87 | 5.22 |
|  |  | LPL | -1.11 | 11.01 | 0.920 | -22.69 | 20.47 |
| Partner focus × attachment anxiety | Pain rating |  | -0.48 | 0.30 | 0.106 | -1.06 | 0.10 |
|  | N1 | LPA | -0.11 | 1.32 | 0.931 | -2.71 | 2.48 |
|  |  | LPL | -3.35 | 4.49 | 0.455 | -12.15 | 5.44 |
|  | N2 | LPA | 0.74 | 1.93 | 0.700 | -3.04 | 4.52 |
|  |  | LPL | 1.86 | 4.57 | 0.684 | -7.09 | 10.81 |
|  | P2 | LPA | -0.97 | 1.80 | 0.589 | -4.50 | 2.56 |
|  |  | LPL | -3.34 | 11.96 | 0.78 | -26.78 | 20.09 |
| Partner focus × attachment avoidance | Pain rating | | 0.32 | 0.21 | 0.133 | -0.10 | 0.73 |
|  | N1 | LPA | 2.00 | 1.33 | 0.134 | -0.61 | 4.61 |
|  |  | LPL | 5.88 | 4.72 | 0.213 | -3.38 | 15.13 |
|  | N2 | LPA | -1.03 | 1.55 | 0.506 | -4.06 | 2.00 |
|  |  | LPL | -0.13 | 3.66 | 0.972 | -7.30 | 7.05 |
|  | P2 | LPA | -0.16 | 1.40 | 0.907 | -2.91 | 2.58 |
|  |  | LPL | -4.94 | 9.31 | 0.596 | -23.18 | 13.30 |
| Attachment anxiety × attachment avoidance | Pain rating | | 0.04 | 0.24 | 0.857 | -0.42 | 0.51 |
|  | N1 | LPA | 0.26 | 0.75 | 0.727 | -1.21 | 1.73 |
|  |  | LPL | -1.69 | 3.56 | 0.634 | -8.66 | 5.28 |
|  | N2 | LPA | 1.26 | 2.84 | 0.658 | -4.30 | 6.82 |
|  |  | LPL | 1.98 | 6.95 | 0.776 | -11.64 | 15.60 |
|  | P2 | LPA | 2.43 | 3.60 | 0.499 | -4.63 | 9.50 |
|  |  | LPL | -5.96 | 19.10 | 0.755 | -43.40 | 31.47 |
| Partner focus × attachment anxiety × attachment avoidance | Pain rating | | -0.20 | 0.18 | 0.260 | -0.54 | 0.15 |
|  | N1 | LPA | -0.06 | 0.76 | 0.938 | -1.54 | 1.43 |
|  |  | LPL | -1.07 | 2.64 | 0.686 | -6.23 | 4.10 |
|  | N2 | LPA | 1.15 | 2.67 | 0.667 | -4.08 | 6.37 |
|  |  | LPL | -6.65 | 6.31 | 0.292 | -19.02 | 5.72 |
|  | P2 | LPA | 1.59 | 2.49 | 0.524 | -3.30 | 6.48 |
|  |  | LPL | 8.69 | 16.57 | 0.600 | -23.79 | 41.18 |

*Note*. LPA = local peak amplitude; LPL = Local peak latency
